# Bile Carriage of *optrA*-Positive Enterococcus faecium in a Patient with Choledocholith

**DOI:** 10.1128/spectrum.02852-22

**Published:** 2023-03-28

**Authors:** Li Deng, Wendong Zhen, Jing Wang, Dachuan Lin

**Affiliations:** a Department of Laboratory Medicine, Shenzhen University General Hospital, Shenzhen University, Shenzhen, China; b Key Laboratory of Prevention and Control of Biological Hazard Factors (Animal Origin) for Agrifood Safety and Quality, Ministry of Agriculture of China, Yangzhou University, Yangzhou, China; c Guangdong Key Laboratory of Regional Immunity and Diseases, Shenzhen University School of Medicine, Shenzhen University, Shenzhen, China; University of California, Davis

**Keywords:** bile, *E. faecium*, *optrA*

## Abstract

We isolated one Enterococcus faecium isolate SZ21B15 from a bile sample of a patient with choledocholith in Shenzhen, China in 2021. It was positive for oxazolidinone resistance gene *optrA* and was intermediate to linezolid. The whole genome of E. faecium SZ21B15 was sequenced by Illumina Hiseq. It belonged to ST533 within the clonal complex 17. The *optrA* gene and additional two resistance genes *fexA* and *erm*(A) were located within a 25,777-bp multiresistance region, which was inserted into the chromosomal *radC* gene, being chromosomal intrinsic resistance genes. The chromosomal *optrA* gene cluster found in E. faecium SZ21B15 was closely related to the corresponding regions of multiple *optrA*-carrying plasmids or chromosomes from *Enterococcus*, *Listeria*, Staphylococcus, and *Lactococcus* strains. It further highlights the ability of the *optrA* cluster that transfers between plasmids and chromosomes and evolves by a series of molecular recombination events.

**IMPORTANCE** Oxazolidinone are effective antimicrobial agents for the treatment of infections caused by multidrug-resistant Gram-positive bacteria, including vancomycin-resistant enterococci. The emergence and global spread of transferable oxazolidinone resistance genes such as *optrA* is worrisome. *Enterococcus* spp. can become causes of hospital-associated infections and are also widely distributed in the gastrointestinal tracts of animals and the natural environment. In this study, one E. faecium isolate from bile sample carried chromosomal *optrA*, being intrinsic resistance gene. *optrA*-positive E. faecium in bile not only makes the treatment of gallstones difficult, but also may become a reservoir of resistance genes in the body.

## OBSERVATION

Linezolid, the first commercially available oxazolidinone, is a crucial antimicrobial agent against infections caused by multidrug-resistant Gram-positive bacteria, including methicillin-resistant Staphylococcus aureus (MRSA), vancomycin-resistant enterococci, and penicillin-resistant pneumococci (1). The emergence and wide spread of linezolid-resistant bacteria is worrying. The multiresistance gene *cfr*, as the first transferable oxazolidinone resistance gene, has been globally reported among Gram-positive and Gram-negative bacteria from different origins, particularly in China (1). In 2015, a novel transferable oxazolidinone resistance gene *optrA*, located on a conjugative plasmid pE349, was identified in Enterococcus faecalis of human origin in China, and further detected in E. faecalis and E. faecium from food-producing animals and humans in China (2). Recently, *poxtA*, as a novel phenicol-oxazolidinone-tetracycline resistance gene, was found in a clinical MRSA in Italy (3). It has been also identified in *Enterococcus* and Staphylococcus isolates from human, food-producing animals and food products (1). Here, we reported the detection of *optrA* in one E. faecium strain from bile of patient with choledocholith.

E. faecium isolate SZ21B15 was obtained from a bile sample collected from a 71-year-old male patient with choledocholith in a tertiary hospital in Shenzhen, China in October 2021. As part of routine surveillance, the oxazolidinone resistance genes (*cfr*, *optrA*, and *poxtA*) were detected as previously described (2, 4, 5) and *optrA* was identified in SZ21B15. MICs of 13 antimicrobial agents were tested by using the broth microdilution method. The results were interpreted by Clinical and Laboratory Standards Institute (CLSI) M100, 30^th^ edition. Gentamicin (≥64 mg/L) was interpreted according to the epidemiological cutoff value for E. faecium set by European Committee on Antimicrobial Susceptibility Testing (EUCAST; https://www.eucast.org/). The isolate SZ21B15 exhibited MIC of 4 mg/L to linezolid, classified as intermediate to linezolid. Similar results were previously observed in *optrA*-positive enterococci (6, 7). It was resistant to erythromycin, chloramphenicol, and ciprofloxacin, and intermediate to nitrofurantoxin, but was susceptible to penicillin, ampicillin, gentamicin, daptomycin, teicoplanin, vancomycin, fosfomycin, and high-level mupirocin (Table S1).

To characterize this *optrA*-positive isolate, we used Illumina Hiseq platform to sequence the whole genome of E. faecium SZ21B15. Genomic DNA extraction, library preparation, and sequencing were performed as previously described (8, 9). A total of 224 contigs (>200 bp) (GenBank accession on. PRJNA861704) were obtained by using SPAdes version 3.15.4 to assemble the raw data. We used the Center for Genomic Epidemiology (CGE) pipeline (https://cge.cbs.dtu.dk/) to analyze the sequence type (ST) and antimicrobial resistance genes. The *optrA*-positive E. faecium strain SZ21B15 belonged to ST533 within the commonly observed clonal complex 17. Thus far, only three E. faecium ST533 isolates were retrieved from PubMLST Enterococcus faecium database (https://pubmlst.org/organisms/enterococcus-faecium) originating from patients (*n* = 2) and the environment (*n* = 1) in Europe.

**FIG 1:**
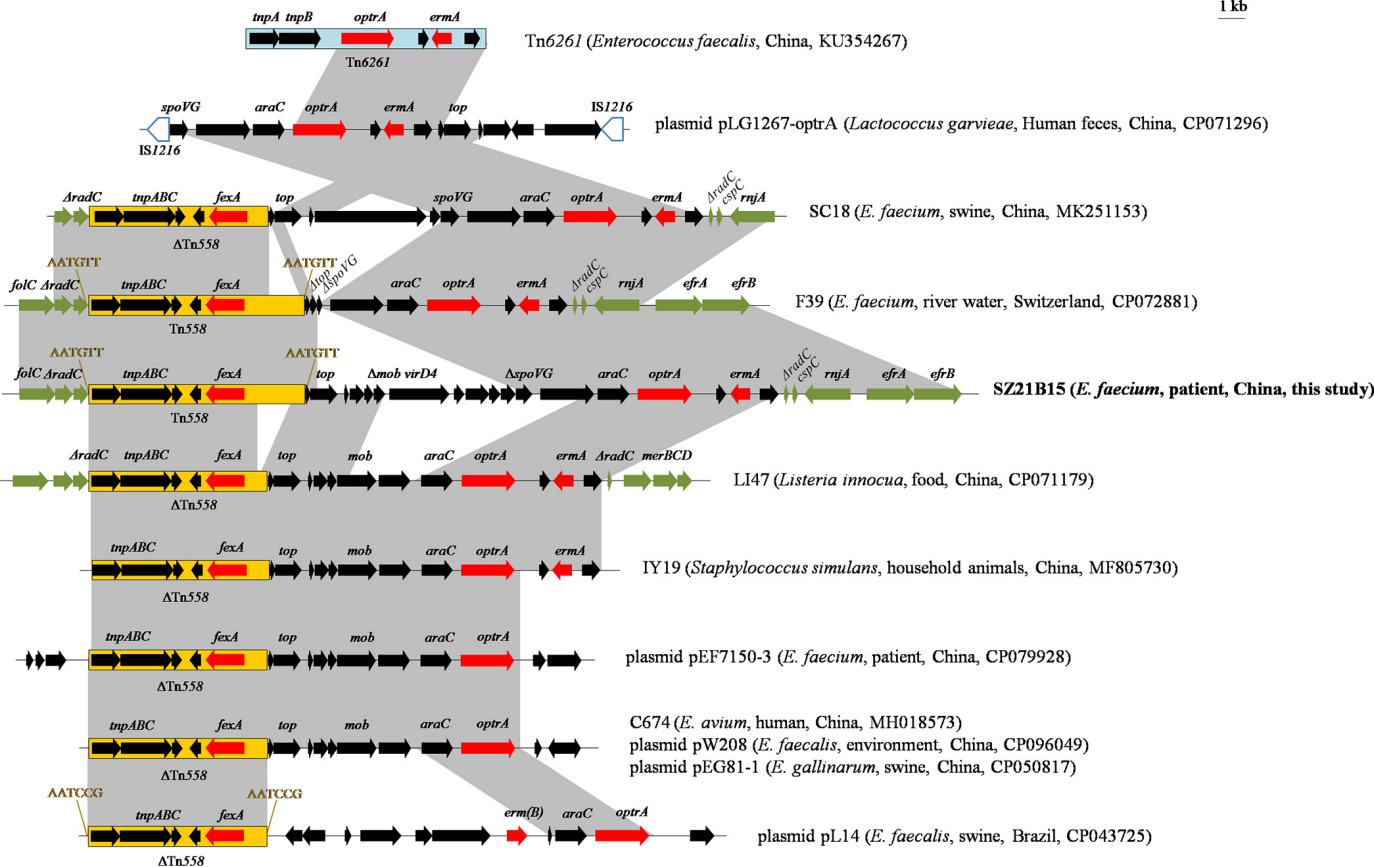
The *optrA* cluster in chromosome of E. faecium strain SZ21B15 in this study and comparison with other *optrA*-positive plasmids or chromosomes. The arrows indicate the positions and transcription directions of the genes. Regions with >99% identity are shaded in gray. Δ indicates a truncated gene or mobile element. Direct repeats are indicated by arrows and sequences.

E. faecium SZ21B15 harbored five resistance genes, including the linezolid resistance gene *optrA*, the phenicol exporter gene *fexA*, the aminoglycoside resistance gene *aac(6’)-Ii*, and the macrolide resistance genes *erm*(A) and *msr*(C). The *optrA* gene and additional two resistance genes [*fexA* and *erm*(A)] was carried by the largest contig (294, 884-bp) corresponding to the chromosome of E. faecium. As shown in Fig. 1, chromosomal *optrA* gene cluster found in E. faecium SZ21B15 was closely related to the corresponding regions of multiple *optrA*-carrying plasmids or chromosomes from *Enterococcus*, *Listeria*, Staphylococcus, and *Lactococcus* strains. The 25,777-bp multiresistance region (MRR) was inserted into the *radC* gene, encoding a DNA repair protein (Fig. 1). Similar insertion of the *fexA*-*optrA*-*erm*(A) region into *radC* was also found in E. faecium strains from pig and river water and Listeria innocua isolate LI47 from food in China (Fig. 1). The chromosomal *radC* gene is a common integration hot spot for Tn*554* family transposons, such as Tn*558* and *optrA*-positive Tn*6674* (10).

An 8,065-bp transposon Tn*558* associated with the *fexA* gene was identified in E. faecium strains SZ21B15 in this study and F39 (water, CP072881) from Switzerland, and was flanked by 6-bp direct repeats (5′-AATGTT-3′). The *fexA* gene within a 1,436-bp shorter Tn*558* was frequently located upstream of *optrA* in multiple plasmids or chromosomes, such as E. faecalis plasmids pW208 (environment, CP096049) and pL14 (swine, CP043725), chromosomal DNA of isolates SC18 (E. faecium, swine, MK251153), LI47 (*L. innocua*, food, CP071179), IY19 (*S. simulans*, animal, MF805730), and C674 (*E. avium*, human, MH018573) (Fig. 1). Tn*558* is suspected to play an essential role in the transmission of *fexA*-*optrA* arrangement (6). The central *optrA* part (Δ*spoVG*-*araC*-*optrA*-*ermA*) was highly similar (>99.9%) to the corresponding regions of chromosomes of E. faecium strains F39 and SC18 and L. garvieae plasmid pLG1267-optrA (CP071296, human), except that a complete *spoVG* was observed in SC18 and plasmid pLG1267-optrA, and a 415-bp shorter Δ*spoVG* was observed in F39 (Fig. 1). The 2,816-bp *araC*-*optrA* segment was present in all *optrA*-carrying plasmids or chromosomes (Fig. 1). A 3,429-bp fragment was located downstream of *araC*-*optrA*, including the macrolide resistance gene *erm*(A) and two putative proteins, which probably originated from transposon Tn*6261* (KU354267) from E. faecalis (Fig. 1).

The 7810-bp region, including gene *top*, an incomplete Δ*mob*, *virD4*, and eight open reading frames encoding hypothetical proteins was located between Tn*558* and the *optrA* part in SZ21B15, and a 2,999-bp segment, including *top*, Δ*mob*, and four putative proteins was identical to the corresponding regions of multiple *optrA*-positive chromosomes or plasmids, e.g., *L. innocua* LI47, *S. simulans* IY19, *E. avium* C674, plasmids pEF7150-3 (E. faecium, patient, CP079928), pW208, and pEG81-1 (*E. gallinarum*, swine, CP050817), although a complete *mob* gene was observed in them (Fig. 1).

The *optrA* gene was first identified in E. faecalis from a human in China and was more frequently in E. faecalis from food-producing animals than from humans (2). Since the discovery of *optrA* in enterococci, it has been successively and globally identified in Gram-positive bacteria such as Staphylococcus, Streptococcus, *Clostridium*, *Lactococcus*, *Listeria,* and Gram-negative pathogen Campylobacter from different sources (1, 11). Various plasmids, transposons, integrative and conjugative elements, prophages, and insertion sequences are responsible for horizontal transmission of *optrA* (1). The chromosomal integration of *optrA* together with additional resistance genes such as *erm*(A) and/or *fexA* has been previously described in E. faecalis and E. faecium from pigs and humans, being chromosomal intrinsic resistance genes (6, 7). Diverse but related *optrA* genetic structures are observed in plasmids or chromosomes from different species, suggesting that the *optrA* cluster may jump between plasmids and chromosomes via mobile elements and evolve by capturing, losing and rearranging molecular modules. Furthermore, *Enterococcus* spp. has become the common bacteria in bile culture with biliary diseases (12). This study is the first report of *optrA* in E. faecium from bile. *optrA*-positive E. faecium in bile not only makes the treatment of gallstones difficult, but also may become the resistance gene pool of other pathogens in the body. It has always been said that the intestine is a reservoir of drug-resistant genes. Our study found that drug-resistant bacteria can also exist in the gallbladder upstream of the intestine. It is worth paying attention to whether drug-resistant bacteria can colonize in the intestine for a long time or transmit resistance genes to other bacteria in the intestine.

### Ethical approval.

This research involving clinical isolates were reviewed and approved by the Ethical Committee of the Shenzhen University Health Science Center (PN202200030).
