# Supplementary material for: Bile Carriage of optrA-Positive Enterococcus faecium in a Patient with Choledocholith
Source: Microbiol Spectr. 2023 Mar 28;11(2):e02852-22. doi: 10.1128/spectrum.02852-22 (PMC10101025; doi:10.1128/spectrum.02852-22)
Supplement: Supplemental file 1 — Table S1. Download spectrum.02852-22-s0001.docx, DOCX file, 0.02 MB [file spectrum.02852-22-s0001.docx]

**Table S1.Results of antimicrobial susceptibility test**

| **Antimicrobial agents** | **MIC (mg/L)** | **Interpretation** |
| --- | --- | --- |
| penicillin | 2 | S |
| ampicillin | ≤2 | S |
| gentamicin | 4 | S |
| daptomycin | 4 | S |
| teicoplanin | ≤1 | S |
| vancomycin | ≤0.5 | S |
| erythromycin | >8 | R |
| chloramphenicol | 32 | R |
| linezolid | 4 | I |
| High-level mupirocin | ≤256 | S |
| fosfomycin | 64 | S |
| nitrofurantoin | 64 | I |
| ciprofloxacin | 4 | R |

S: sensitive; I: intermediate; R: resistant.
